# Evaluation of MRI protocols for the assessment of lumbar facet joints after MR-guided focused ultrasound treatment

**DOI:** 10.1186/s40349-016-0057-8

**Published:** 2016-04-06

**Authors:** Roland Krug, Loi Do, Viola Rieke, Mark W. Wilson, Maythem Saeed

**Affiliations:** Department of Radiology and Biomedical Imaging, School of Medicine, University of California San Francisco, 185 Berry Street, Suite 350, Campus Box 0946, San Francisco, CA 94107-5705 USA

**Keywords:** MRgFUS, Facet joint, T2-weighted FSE, Delayed contrast-enhanced MRI

## Abstract

**Background:**

MR-guided focused ultrasound (MRgFUS) might be a very safe and effective minimally invasive technique to treat facet joint pain caused by arthritis and other degenerative changes. However, there are still safety concerns for this treatment and challenges regarding MR imaging and temperature mapping due to susceptibility effects between the bone and soft tissue near the joint, which has resulted in poor MR image quality. The goal of this research was to evaluate multiple magnetic resonance imaging (MRI) pulse sequences for characterizing ablated lumbar facet joint lesions created by high-intensity focused ultrasound (FUS) and compare the findings to histological tissue assessment. In particular, we investigated the use of T2-weighted MRI to assess treatment effects without contrast administration.

**Methods:**

An IACUC approved study (*n* = 6 pigs) was performed using a 3T widebore MRI system equipped with an MRgFUS system. Facet joints of the lumbar vertebra were ablated using 1-MHz frequency and multiple sonication energies (300–800 J). In addition to T2-weighted MRI for treatment planning, T1-, T2-, and T2*-weighted and perfusion MRI sequences were applied. Signal intensity ratios of the lesions were determined. Histopathology was used to characterize cellular changes.

**Results:**

Ablation of the facet joint, using MRgFUS, was successful in all animals. T2-weighted images showed high signal intensity in the edematous facet joint and adjacent muscle, while delayed contrast-enhanced T1-weighted images showed an enhanced ring surrounding the target volume. T2*-weighted GRE images revealed inconsistent lesion visualization. Histopathology confirmed the presence of cellular coagulation (shrinkage), extracellular expansion (edema), and hemorrhage in the bone marrow.

**Conclusions:**

MRgFUS provided sufficient precision and image quality for visualization and characterization of ablated facet joints directly after ablation. MRI may help in monitoring the efficacy of FUS ablation without contrast after treating patients with back pain.

## Background

Interest in MR-guided focused ultrasound (MRgFUS) has increased tremendously over the past few years. In MRgFUS, focused ultrasound beams are combined with real-time magnetic resonance imaging (MRI) monitoring to perform controlled thermal ablation [1]. This combined modality is well suited for targeting, characterizing, and quantifying pathologic tissues [2–4]. MRgFUS has recently emerged as an effective treatment option for ablating uterine fibroids [5–10], bone metastases [11], breast cancer [12], hepatocellular carcinoma [13], brain cancer [14], and treating brain cancer by opening the blood-brain barrier [15]. Recently, MRgFUS has also been applied to the treatment of facet joints in patients with lower back pain and bone metastasis [16, 17]. Significant improvement in patient lifestyle was evident after treatment [17].

In MRgFUS, image guidance is extremely important for both treatment planning and real-time temperature monitoring. MRI can provide instant information on temperature changes in tissues [18] that plays an important role in treatment planning and tissue necrosis. It also ensures safety monitoring of the procedure and energy deposition. Although much attention has been paid to the optimization of thermometry approaches for MRgFUS [19, 20], less focus has been placed on the optimization of MR protocols for characterization of ablated tissues after treatment. In particular, imaging techniques for lesion detection without the need of contrast would allow potential repetitions of focused ultrasound (FUS) treatment within the same session. However, this is not possible once contrast has been administered [21]. Hijnen et al. [22] investigated several effects of contrast agent before FUS treatment. They found a significant frequency shift due to local magnetic field from the contrast agent, which resulted in wrong calculations for temperature and dose. Furthermore, short-term trapping of contrast in the coagulated tissue volume was observed, but no effect on long-term retention was found. Thus, lesion visualization without the need of contrast would be an important progress for clinical applications of MRgFUS. Harnof et al. recently showed that visualization and characterization of facet joint lesions after thermal ablation is particularly challenging with very limited MR image quality [16]. Thus, the goal of this study was to further optimize and evaluate multiple MRI sequences for characterizing ablated lumbar facet joint created by FUS.

## Methods

### Animal preparation

All experimental procedures received approval from the Institutional Animal Care and Use Committee (IACUC). Six healthy female farm pigs (Pork Power Farms, Turlock, CA) were premedicated with 0.5 mg/kg acepromazine (PromAce; Fort Dodge Animal Health, Fort Dodge, IA) and 30 min later 25 mg/kg ketamine (Ketaset; Fort Dodge Animal Health). The pigs were then anesthetized with a mixture of isoflurane 2–5 % and oxygen. Saline (10 mL/kg/h) was intravenously infused throughout the experiment for hydration. Vital signs (heart rate, electrocardiogram (ECG), respiratory rate, O_2_-saturation) were monitored throughout the procedure. To minimize muscle twitching and diaphragm motion during inspiration and expiration in anesthetized pigs, rocuronium (0.1 mg/kg) was intravenously administered prior to ablation.

### MRgFUS setup and treatment

All FUS experiments were performed using the ExAblate 2000 System (InSightec Ltd., Tirat Carmel, Israel) with a phased array transducer of 208 elements embedded in the MR scanner table. The table was connected to a 3T MRI widebore scanner (see details below). The skin around the targeted treatment areas was shaved for each animal, cleaned, and closely examined for any skin defects or scars, which might impede the propagation of acoustic energy from the transducer. Each pig was then placed onto a gel pad on the scanner table in a supine position, inside a shallow bath filled with degassed water.

**Fig. 1:**
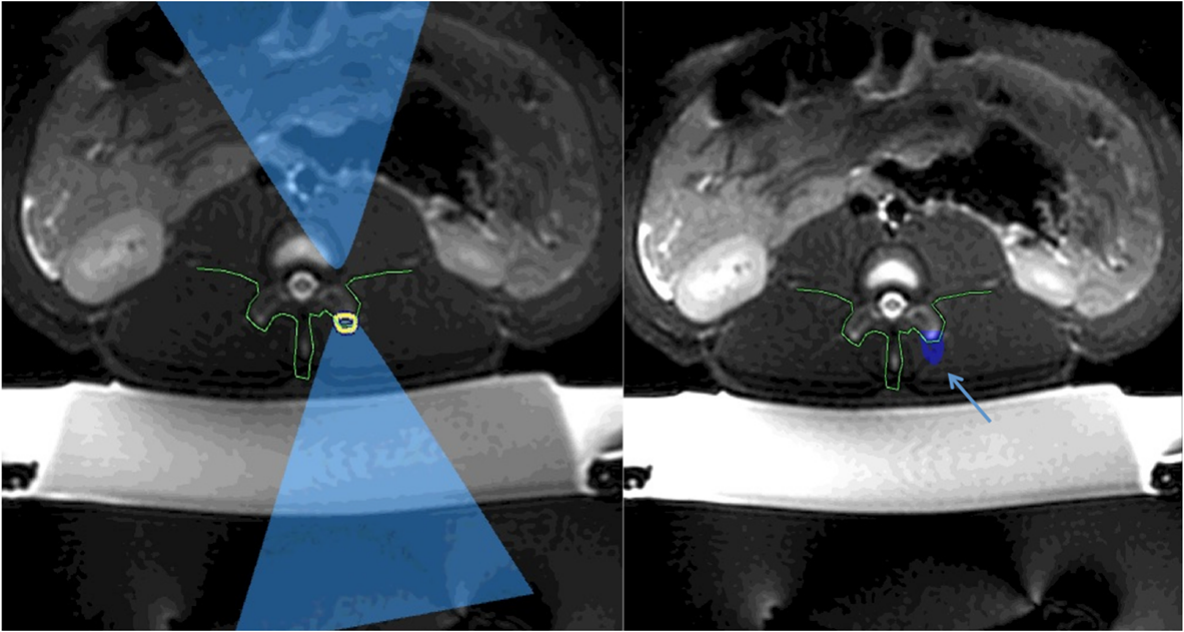
T2-weighted planning images are shown with the focal spot on the facet joint (*left image*). The beam orientation was chosen to best protect the spinal nerve roots, the spinal canal, and the spinal process as outlined in *green*. After HIFU treatment, the area of ablation is shown in *blue* (dose overlay) on the right image

A three-plane localizer was performed to verify adequate positioning relative to the transducer. T2-weighted treatment planning sequences were added. Adequate coverage of all treated facet joints was obtained with 3-mm slice thickness. The planning images were then transferred to the ExAblate workstation where the target bone was segmented (Fig. 1) and the skin surface was identified. Low-energy test sonications were performed in the muscles far away from the target in order to calibrate the FUS beam location. Three to four lumbar facet joints (L3/4-L6/7) on the left side of the animal were ablated per pig using an ultrasound frequency of 1 MHz and the following spot energies: 300 J (duration = 25 s, acoustic power = 15 W, cooling duration = 25 s), 500 J (duration = 25 s, acoustic power = 20 W, cooling duration = 25 s), 650 J (duration = 25 s, acoustic power = 25 W, cooling duration = 25 s), and 800 J (duration = 25 s, acoustic power = 32 W, cooling duration = 25 s). The energies were selected in order to cover the range used in previous publication for facet joint ablation [16, 17].

We have treated one side of three to four vertebral levels, thus three to four facet joints per animal. We have identified a central MRI slice from the planning images of each facet joint and added one more slice on each side for the whole treatment volume. We have applied two partially overlapping sonication on each slice resulting in a total of six sonications per joint. The temperature was assessed during each sonication using echo planar imaging (EPI) as previously described [20, 23]. The temperature rise was measured at the focal point and in the far field adjacent to the intervertebral foramen.

### MR imaging

All imaging was performed on a 3T widebore scanner (Discovery MR 750w, GE Healthcare, Waukesha, WI). For signal acquisition, the default body coil was used and a 64 channel receive only cardiac coil (GE Healthcare, Waukesha, WI). In addition to T2-weighted MRI for the treatment planning, several MR images were acquired before and immediately after FUS treatment (see Table 1 for imaging parameters). The pulse sequences included axial, sagittal, and coronal 2D T1-weighted fast spin echo (T1-FSE), a 2D T2-weighted FSE with fat saturation (T2-FSE), and 2D T2*-weighted gradient recalled echo (T2*-GRE). First-pass perfusion was performed using axial 2D gradient echo with FOV = 28 cm, slice thickness = 5 mm, and a matrix size of 96 × 96 voxels. T2*-GRE and T2-FSE were performed before and after FUS application but before contrast injection. T1-FSE was performed before and after FUS application as well as after contrast injection. Bolus injection of 2 mmol/kg gadolinium diethylenetriamine pentaacetate (Gd-DTPA) was delivered after the FUS treatment for perfusion and delayed contrast enhancement (DCE) imaging after treatment.

**Table 1:** MR sequences and imaging parameter

Pulse sequence	TR (ms)	TE (ms)	ETL	rBW (kHz)	Flip angle (°)	Slice (mm)	In-plane resolution (mm)	Scan time
T1-FSE	600–800	6.5	6	31.25	90	3–5	1–1.5	3’13”
T2-FSE	4500–8300	68	12	15.63	90	3–5	1–1.5	4’01”
T2*-GRE	1550	15	N.A.	19.23	30	4	1–1.5	5’04”
Perfusion	3.7	1.5	N.A.	62.5	9	5–7	2.9	4’57”

The scan times varied according to coverage and number of slices for the different orientations

*N.A.* not applicable

### Image and statistical analysis

On all acquired images, the patterns of delineation of the treated facet joints were compared to the untreated contralateral joints. Signal to noise (SNR) was evaluated as the mean signal intensity (SI) in the region of interest divided by the standard deviation of the noise, while SI ratio (SIR) was determined by dividing SI of ablated lesion over surrounding normal tissues. Thus, SIR <1 was defined as hypointense, SIR = 1 as isointense, and SIR >1 as hyperintense. Contrast to noise ratio (CNR) was evaluated by the difference in signal between two regions divided by the standard deviation of the noise. Images were assessed for the presence of edema on the T2-FSE images as well as loss of cellular and vascular integrity on DCE T1-FSE and T2*-GRE images. SI(s) as a function of time were also measured in ablated and surrounding normal tissues on perfusion images. Paired Student’s *t* test was used for statistical analysis. Data were presented as mean ± SEM and a *P* value less than 0.05 was considered significant.

### Histology

Animals were heparinized prior to euthanasia. All animals were sacrificed ~4 h after ablation by IV injection of saturated potassium chloride. Formalin (4 %) was infused for 1 h to fix the tissue in situ. Tissue samples (treated and contralateral non-treated facet joints and paravertebral muscles) were obtained, sliced, and fixed in 70 % formalin. Microscopic sections (5 μm) were stained with hematoxylin and eosin stain (H&E) and examined microscopically. Histopathological sections through the paravertebral muscles, dorsal root ganglion, and facet joint were acquired.

## Results

The instant temperature changes were monitored during treatment using the standard proton resonance frequency (PRF) method with echo planar MRI [20]. The mean temperature during sonication was 62.7 ± 9.8 °C. After the procedure, the capability of each pulse sequence to depict the ablated facet joints and adjacent tissue was determined. Table 1 shows the differences in scan time and spatial resolution between the used pulse sequences. Our study showed that the image quality from the body coil was sufficient to clearly visualize the lesions. We found a gain in SNR of a factor of 1.7 by switching the body coil to a 64 channel cardiac coil, but this required changing the table and position of the animal and may lead to misregistration of the images.

**Fig. 2:**
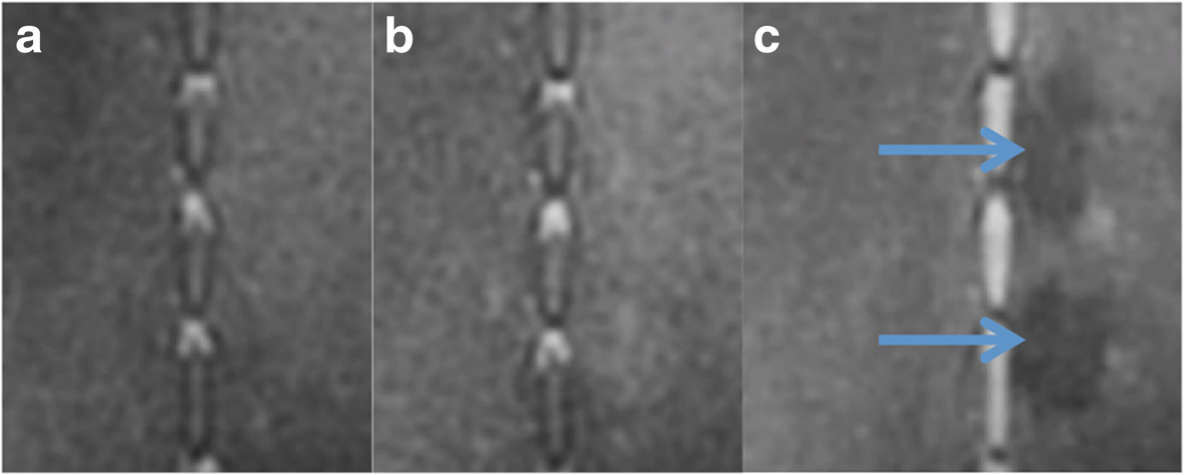
Coronal T1-FSE images. **a** Baseline. **b** After FUS ablation. **c** After both FUS and contrast injection. The lesions were delineated, as hypoenhanced zone, after administration of 0.15 mmol/kg Gd-DTPA, suggesting lack of delivery of the contrast media due to the damage of microvessels

**Fig. 3:**
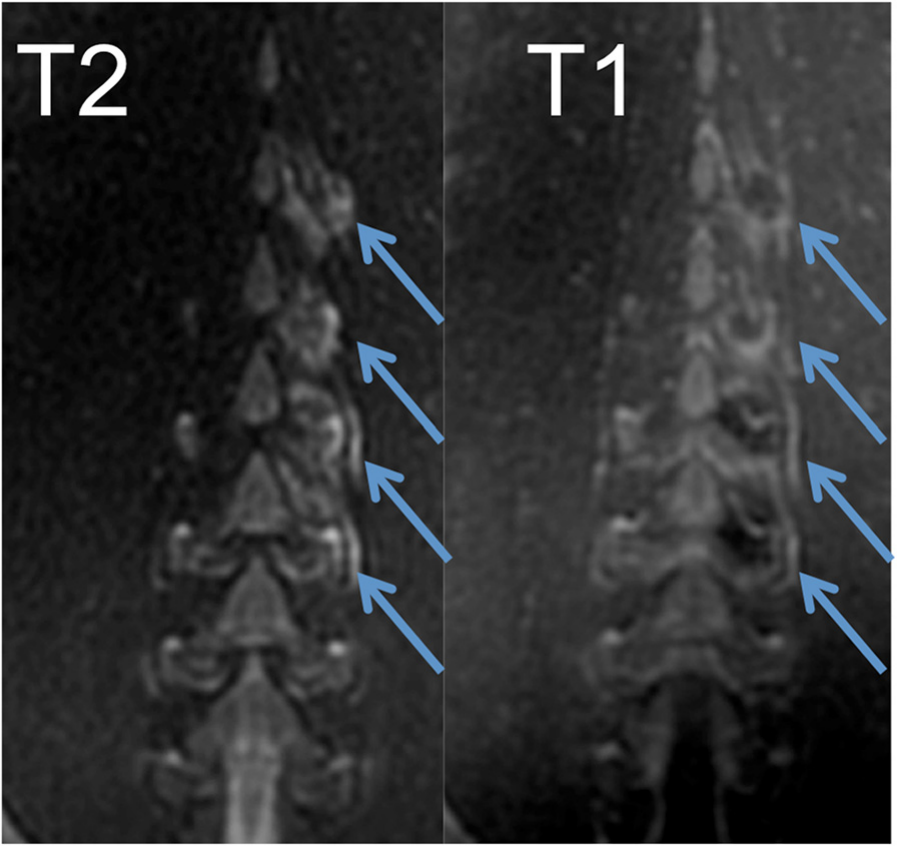
Coronal T2-FSE (*left*) and DCE T1-FSE (*right*) after FUS treatment. The T2-FSE image shows a positive contrast of the lesions. The DCE T1-FSE image shows the hyperenhancement of the border zone, but not the core of ablated lesion. The following energies were applied (from *top* to *bottom*): 650, 300, 500, and 800 J, and the *arrows* indicate ablated lesions. A positive relationship between energy and lesion size can be appreciated in both images

**Fig. 4:**
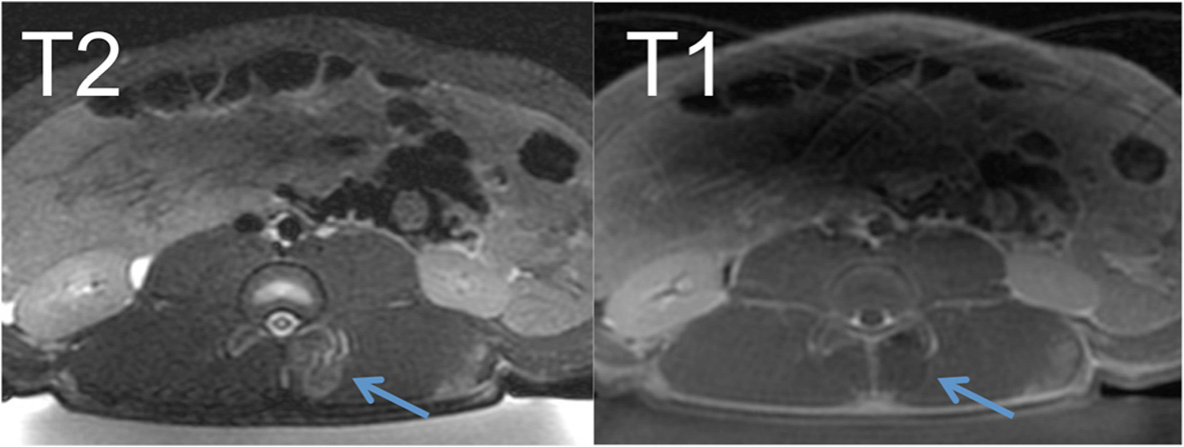
Axial T2-FSE (*left*) and DCE T1-FSE (*right*) after FUS treatment. The T2-FSE image shows a positive contrast of the lesions. The DCE T1-FSE image shows the hyperenhancement of the border zone, but not the core of ablated lesion

T1-FSE failed to provide sufficient contrast between ablated and adjacent normal tissues prior to administration of contrast media (Fig. 2). Gd-DTPA delineated the ablated region by enhancing the signal at the border zone (hyperemia), suggesting that the diffusion of contrast media was limited due to vascular damage. After contrast administration, all lesions were depicted with negative contrast to the surrounding tissue on T1-FSE (Figs. 2, 3, and 4) and demonstrated focal ovoid hypoenhancement (SIR = 0.8) of the bone and soft tissue at the target volume with a thin rim of hyperenhancement (SIR = 1.6). These findings confirmed the presence of necrosis and vascular damage that led to the exclusion of Gd-DTPA from entering to the core of the lesion.

**Fig. 5:**
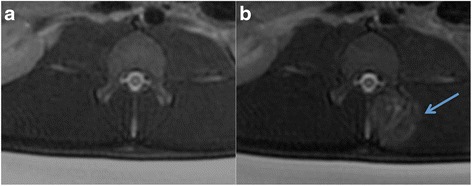
Axial T2-weighted images. **a** Acquired before FUS. **b** After FUS treatment. The lesion is clearly visible as hyperintense signal, suggesting the presence of edema. The sonication energy used for the depicted joint was 800 J. Smaller lesion sizes were generated with smaller sonication energies

On T2-FSE (Figs. 3, 4, and 5), the lesions were clearly shown as hyperintense (SIR = 3.9) after ablation but before contrast injection, suggesting the presence of interstitial edema. Although SNR was in general higher on post-contrast DCE T1-FSE, CNR between the lesions and the normal tissue was factor 1.8 higher on T2-FSE.

**Fig. 6:**
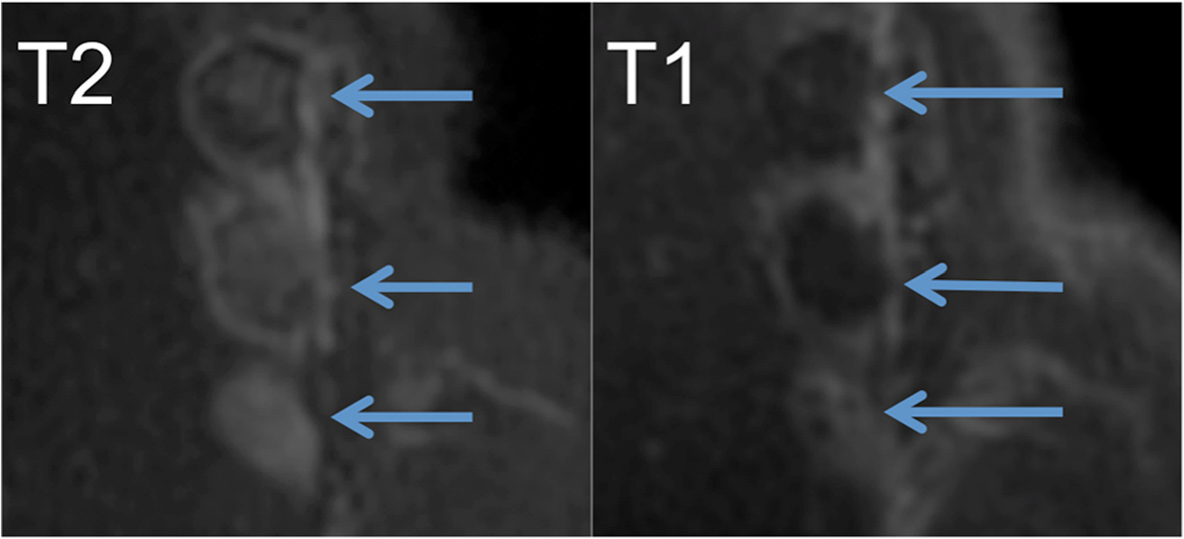
Sagittal MR images of the paraventricular muscles obtained on T2-FSE (*left*) and DCE T1-FSE. T2-FSE image shows the hyperintense edematous ablated lesion, while T1-FSE image shows the donut pattern enhancement after administration of Gd-DTPA

For comparisons, additional sonications were performed in the skeletal muscle adjacent to the facet joint and paravertebral muscles. Again, the interstitial edema can be appreciated on T2-FSE where the lesions are shown as hyperintense (Fig. 6) compared to surrounding tissue in all lesions (SIR = 1.90 and SIR = 2.50). DCE T1-FSE showed again focal hypoenhancement and surrounding hyperemia.

**Fig. 7:**
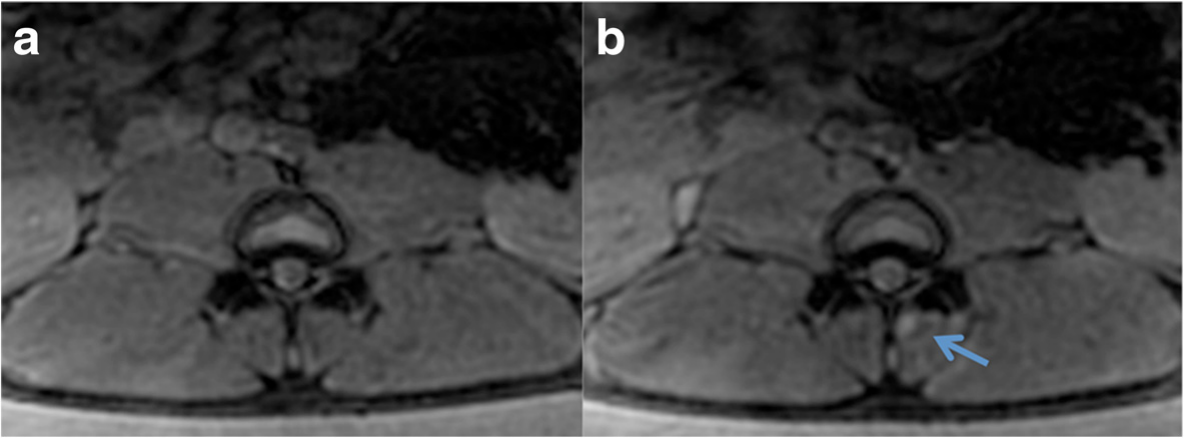
Representative axial T2*-weighted images acquired before (**a**) and after (**b**) FUS ablation. The slice location corresponds to the slices depicted in Fig. 4. A small hyperintense lesion is visible after ablation (*arrow*)

T2*-GRE images acquired after sonication showed small, inconsistent hyperintense (SIR = 1.5) zones close to the treated area. This was not observed at the contralateral joints (Fig. 7). These hyperintense zones appeared smaller than the edematous regions on T2-FSE images.

A lack of differential contrast between the ablated facet joint or paravertebral muscles and normal adjacent tissues was found on first-pass perfusion images, which can be attributed to a trade-off between time resolution, spatial resolution, number of covered slices, and slice thickness.

**Fig. 8:**
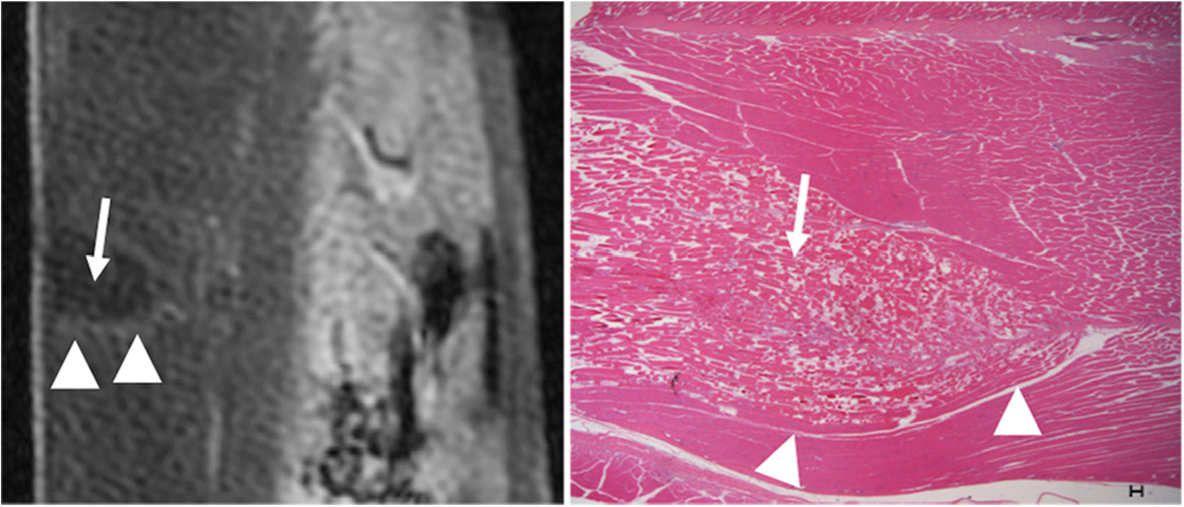
Sagittal image and histopathological section through the paravertebral muscles show the wedge shape-ablated lesion on DCE (*left*) and microscopy (*right*). *Arrows* denote the necrotic core with damaged tissue. Note the border edematous hyperenhanced zone (*arrowheads*) surrounding the ablated core on DCE (scale shows the SI range in arbitrary units). The border edematous zone is also seen on microscopy (*arrowheads*, calibration bar = 100 μm)

**Fig. 9:**
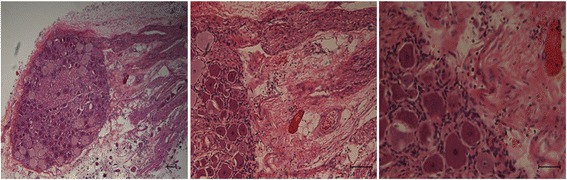
Microscopic sections of dorsal root ganglion after 800-J sonication showing no evidence of injury in neural cells or axons. Magnifications are ×10, ×40, and ×100

**Fig. 10:**
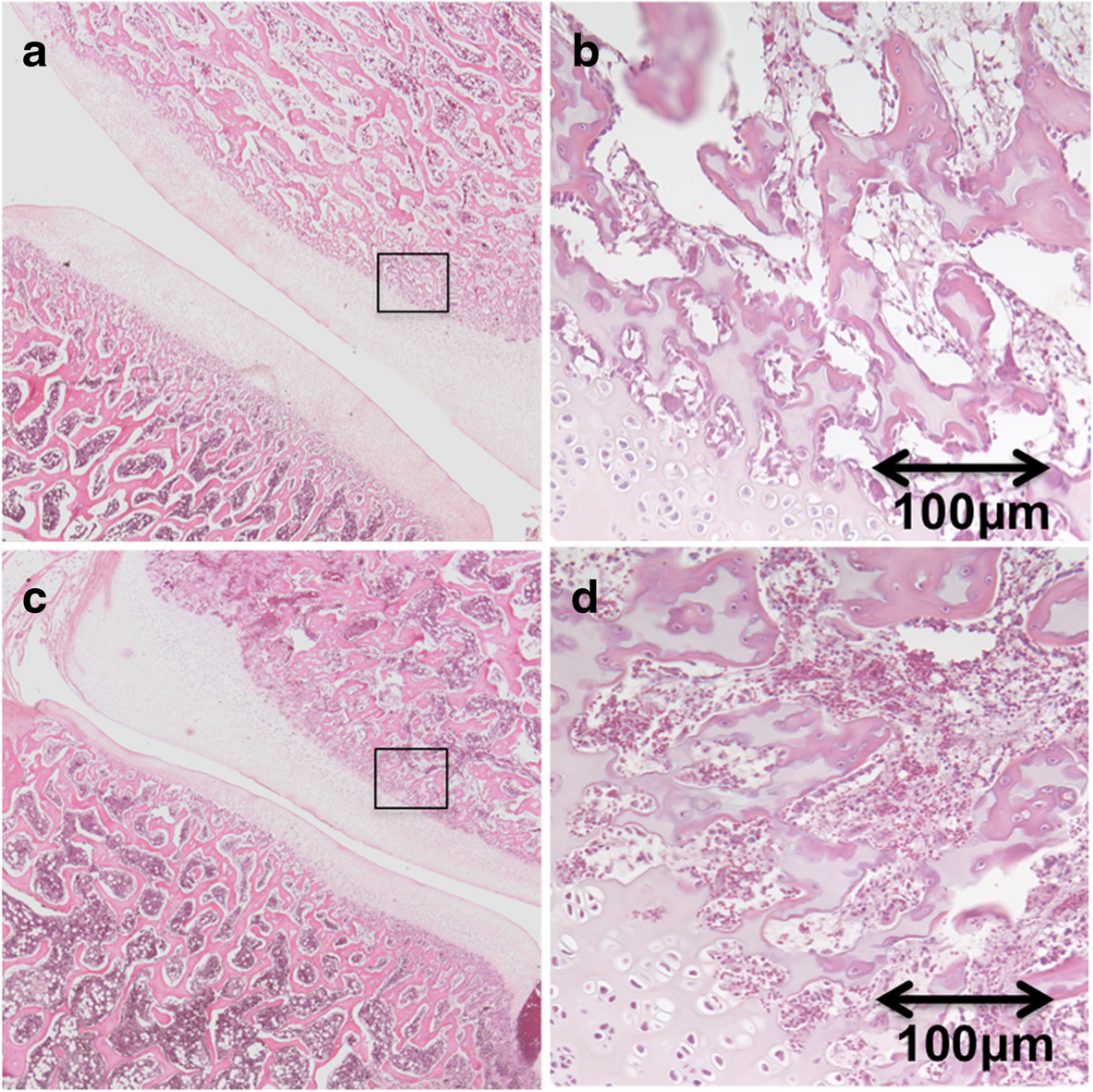
Histological sections of facet joint. **a** Control side with zoom ×2. **b** Control side with zoom ×40. **c** Ablated side with ×2. **d** Ablated side with ×40. Evidence of hemorrhage is clearly demonstrated in the treated facet joint. There was no change in the architecture of the osteoblast, most likely due to the short period of exam after ablation

Microscopic examination confirmed the locations of the ablated tissue, which include coagulation necrosis (shrinkage of the cells) and expansion of extracellular space (interstitial edema) (Fig. 8). No evidence of damage was found in the dorsal root ganglion or the spinal cord as a result of thermal ablation (Fig. 9). Evidence of hemorrhage was seen in the treated facet joint (Fig. 10). But there was no change in the architecture of the osteoblast, most likely due to the short period of exam after ablation.

## Discussion

Using MRgFUS, the ideal imaging protocol should have the following two characteristics. First, it should be highly sensitive to acute tissue changes to allow for accurate evaluation of the treatment. Second, it should allow repetitions of the treatment if necessary. The later demand would exclude the use of contrast agents. The goal of this work was to present a comprehensive study for MRgFUS treatment of the facet joint investigating several MR pulse sequences, sonication energies, and histological findings. Compared to a previous study by Harnof et al. [16], we have further enhanced MR imaging using T1-FSE with and without contrast, T2-FSE, T2*-GRE, and perfusion sequences and confirmed outcomes by histology. One of the major findings of our study is that T2-FSE MRI can provide excellent post-FUS lesion detection without the need of contrast administration. This has important implications for the FUS procedure because the ablation can be immediately evaluated and retreated if necessary. This is not possible once contrast has been administered.

Both pre-contrast T2-FSE and post-contrast DCE T1-FSE sequences have the potential to delineate ablated facets and surrounding tissues. Microscopic examination of biopsies confirmed the thermal effects on tissues adjacent to the facet joints and lumbar vertebrae. T2-FSE showed strong positive contrast with hyperintense lesion signal suggesting the presence of edema in the lesion. In addition, slightly increased blood flow (hyperemia) could also be observed in the peripheral zones similar to DCE (Figs. 3, 4, and 5). Although the scan time was slightly increased on T2-FSE scans (Table 1), there was no need for administration of contrast media or delay time (typically 10 min) for imaging. Thus, this might be a fast approach for lesion detection and verification immediately after treatment. We found that a 3-mm slice thickness and 192 matrixes provided the best trade-off between image quality, contrast, and scan time for all sequences (Table 1). Previously, inconsistent results have been reported using T2-weighted images, e.g., heterogeneous appearance was shown after FUS in uterine fibroids [24], prostate [25], and pancreatic tumors [26].

In addition to T2-FSE and DCE, T2*-GRE might be useful to distinguish edema from hemorrhage in ablated lesions. T2*-GRE images inconsistently showed small hyperintense lesions that were visible adjacent to the treated joint. It has previously been shown that T2*-GRE is limited in detecting interstitial edema [27]. Due to a trade-off between time resolution, spatial resolution, coverage, and slice thickness, we were unable to obtain meaningful perfusion results. Despite the above limitations, this study provides a basis for further development and standardization of the different techniques. A previous study indicated that the mechanism of loss of vascular integrity during MRgFUS ablation is unclear [28]. Hynynen et al. [1] indicated that noninvasive vascular occlusion could be achieved using thermal MRgFUS, and Wu et al. [29] suggested that the cause of perfusion deficits after FUS ablation is vascular damage. Our histological study indicated that the skeletal muscle showed coagulation necrosis and damaged microvessels after FUS treatment. The damaged vessels with coagulated blood most likely played a critical role in preventing blood perfusion and delivery of MR contrast media to the core of the ablated lesion.

This study showed further that thermal ablation causes shrinkage of the cells and expansion of extracellular space. The expansion of extracellular space is unrelated to extravasation of albumin from the vascular compartment, because extracellular Gd-DTPA was unable to diffuse to the core of the lesions. A dynamic relationship exists between the extracellular and intracellular water. Water molecules exchange passively between intravascular, interstitial, and intracellular compartments under normal circumstances. Water volume in each compartment is determined by mechanisms controlling intracellular osmotic pressure, including ion homeostasis and metabolism, and water transport through sarcolemmal channels and membrane diffusion. Regulation of intracellular pH is an important element in osmotic regulation. ATP-dependent Na^+^/K^+^ pump and intracellular proteins maintain the balance between intra- and extracellular water [30]. All these elements can be influenced by energy metabolism. It has been shown that brief myocardial ischemia leads to energy deficiency, sodium overload, cell swelling, and water dissociation from proteins because of lactate-induced acidosis, thereby increasing the intracellular fraction of free unbound water [31, 32]. We have also looked at treatment effects on bone. Although there was evidence of hemorrhage in the treated facet joint, no change in the architecture of the osteoblast was found. As these effects need more time to manifest, a longitudinal study is clearly warranted.

During FUS treatment, there should be no RF coil in the field of the sonication. Thus, the default body coil needs to be used for treatment planning but could potentially be replaced after treatment. However, we found that the default body coil of the MR system provided sufficient image quality to characterize regional tissue changes (edema, viability) after treatment. Although switching from the default body coil to a phased array coil after FUS treatment provided better SNR, it did not improve lesion detection but was time-consuming and compromised image registration.

The rise in temperature was used to define the target and may not reflect the actual temperature in the target. Furthermore, PRF-based MRI temperature mapping is very sensitive to off-resonance effects near tissue interfaces such as between the bone and muscle and may thus be inconsistent and not reliable near the facet joint. Thus, calibration is usually done within the muscle tissue, and this might be a problem in patients with severe facet arthritis where it might be very difficult to find a homogenous muscle layer. However, since lethal cell damage occurs when temperatures >55 °C are maintained for longer than a second [33], accurate temperature measurements are crucial to monitor the treatment and to evaluate treatment outcomes. Furthermore, post-sonication MRI could potentially be a helpful marker to identify necrotic areas where these high temperatures were met (see Fig. 8). These needs to be further investigated.

The ultimate goal of using MRgFUS for facet joint ablation is to produce lasting pain relief to the patient. Recently, Weeks et al. demonstrated the in vivo applicability of MRgFUS for facet joint palliation with relatively small energies (average 600 J) [17]. They reported no improvements in pain scores in 40 % of the patients most likely due to the low energies used. Very recently, another group from Europe presented preliminary results from a clinical study with 35 patients where they used sonication energies of up to 2300 J [34]. They reported no adverse events and 28 patients had a significant reduction in pain. These results suggest that there is a trade-off between safety and efficacy of MRgFUS and higher sonication energies might be more efficient. However, safety becomes increasingly a concern with higher energies, and clearly, more animal studies are warranted to investigate the optimal treatment strategy while ensuring patient safety.

## Conclusions

In conclusion, we have evaluated several MRI pulse sequences for the assessment of FUS treatment and verified the findings with histology. We found that T2-FSE provides good image quality with positive contrast between the ablated lesion and surrounding tissues and could reduce the imaging cost and overall treatment time, as no contrast agent is needed and thus continuous FUS treatment would be possible. T1-FSE, with the aid of MR contrast media, delineated the borders of ablated lesions and provided evidence of vascular obstruction. Demonstration of perfusion deficit on first-pass perfusion imaging is challenging in the facet joints and needs further improvement. Microscopy revealed the cellular and vascular changes after ablation and provided direct evidence on the causes of hyperintensity on T2-FSE and hyperenhancement on DCE T1-FSE images. In summary, we have presented acute findings in the facet joint after FUS ablation. However, many changes on the tissue level need more time to manifest. Thus, the next important step of this research would be a longitudinal study to investigate changes over time.
